# Improved intratumoral penetration of IL12 immunocytokine enhances the antitumor efficacy

**DOI:** 10.3389/fimmu.2022.1034774

**Published:** 2022-10-27

**Authors:** Keunok Jung, Sojung Yoo, Jung-Eun Kim, Wook Kim, Yong-Sung Kim

**Affiliations:** ^1^ Department of Allergy and Clinical Immunology, Ajou University School of Medicine, Suwon, South Korea; ^2^ Department of Molecular Science and Technology, Ajou University, Suwon, South Korea

**Keywords:** immunocytokine, IL12, solid tumor, tumor penetration, binding kinetics, T cell activation

## Abstract

Tumor-targeting antibody (Ab)-fused cytokines, referred to as immunocytokines, are designed to increase antitumor efficacy and reduce toxicity through the tumor-directed delivery of cytokines. However, the poor localization and intratumoral penetration of immunocytokines, especially in solid tumors, pose a challenge to effectively stimulate antitumor immune cells to kill tumor cells within the tumor microenvironment. Here, we investigated the influence of the tumor antigen-binding kinetics of a murine interleukin 12 (mIL12)-based immunocytokine on tumor localization and diffusive intratumoral penetration, and hence the consequent antitumor activity, by activating effector T cells in immunocompetent mice bearing syngeneic colon tumors. Based on tumor-associated antigen HER2-specific Ab Herceptin (HCT)-fused mIL12 carrying one molecule of mIL12 (HCT-mono-mIL12 immunocytokine), we generated a panel of HCT-mono-mIL12 variants with different affinities (*K*_D_) mainly varying in their dissociation rates (*k*_off_) for HER2. Systemic administration of HCT-mono-mIL12 required an anti-HER2 affinity above a threshold (*K*_D_ = 130 nM) for selective localization and antitumor activity to HER2-expressing tumors versus HER2-negative tumors. However, the high affinity (*K*_D_ = 0.54 or 46 nM) due to the slow *k*_off_ from HER2 antigen limited the depth of intratumoral penetration of HCT-mono-mIL12 and the consequent tumor infiltration of T cells, resulting in inferior antitumor activity compared with that of HCT-mono-mIL12 with moderate affinity of (*K*_D_ = 130 nM) and a faster *k*_off_. The extent of intratumoral penetration of HCT-mono-mIL12 variants was strongly correlated with their tumor infiltration and intratumoral activation of CD4^+^ and CD8^+^ T cells to kill tumor cells. Collectively, our results demonstrate that when developing antitumor immunocytokines, tumor antigen-binding kinetics and affinity of the Ab moiety should be optimized to achieve maximal antitumor efficacy.

## Introduction

Several cytokines, including interleukin 12 (IL12), are key mediators of innate and adaptive antitumor immunity and thus have great potential for cancer therapy (1). However, the systemic administration of recombinant cytokines in clinical settings has been limited by their pleiotropic actions, causing off-target undesirable effects, and the short serum half-life, requiring frequent dosing, resulting in dose-limiting toxicities (2, 3). To overcome these barriers, cytokines are often fused with a tumor-targeted antibody (Ab), referred to as immunocytokines, which can prolong the serum half-life and achieve targeted delivery into the tumors for inducing local anticancer immune responses in the tumor microenvironment (TME) while reducing systemic toxicity (1–3). Although several immunocytokines are in early-stage clinical trials against solid tumors (3), none has been approved to date. Among the key limiting factors to be resolved for achieving favorable clinical outcomes of immunocytokines in the treatment of solid tumors are the poor tumor distribution and limited depth of intratumoral penetration of immunocytokines in tumor tissues due to the abnormal physiological and physical properties of solid tumors (4, 5). Previous reports using tumor-targeted Abs have shown that antigen-binding kinetics and affinity as well as antigen metabolic turnover are critical factors in determining the tumor localization and depth of intratumoral penetration (6). An exceedingly high-affinity Ab tends to penetrate only a few cell layers outside the blood vessels due to tight antigen binding and/or depletion by antigen-mediated internalization and lysosomal degradation before its dissociation from the antigen on cells, thereby limiting the intratumoral diffusion (6, 7). This concept is known as the binding-site barrier (7), which explains the poor tumor tissue penetration and spread of a high-affinity Ab compared with those of lower-affinity Ab in solid tumors (8–10). Likewise, tumor-targeted Ab-based immunocytokines would face the same challenges for solid tumors. A systemically administered immunocytokine needs to be selectively localized in tumors and then diffuse into tumor tissues across the interstitial space to reach as many immune effector cells as possible for their expansion and activation to exert antitumor immunity. Thus, efficient intratumoral penetration of an immunocytokine would be pivotal to stimulate the effector function of intratumoral immune cells within the TME. However, this issue has not been well-investigated to date and is often overlooked when developing anticancer immunocytokines.

In this study, we sought to determine how the tumor antigen-binding kinetics and affinity of immunoglobulin G (IgG) Ab-fused IL12 immunocytokines affect their localization and intratumoral penetration in solid tumors, and thus the antitumor activity through activation of effector T cells within the TME, using murine solid tumor models. IL12 is a heterodimeric cytokine (70 kDa) composed of two disulfide-linked p35 and p40 subunits (11). IL12 signals by monovalent binding to a heterodimeric IL12 receptor (IL12R) comprising IL12Rβ1 and IL12Rβ2 subunits (12). IL12 stimulates the proliferation and cytotoxicity of CD8^+^ T cells and natural killer cells *via* the induction of cytotoxic enzymes and cytokines, mainly interferon-γ (IFNγ), to kill tumor cells (12). Further, IL12 promotes CD4^+^ T helper 1 cell differentiation by augmenting IFNγ production (11). Thus, IL12 is a potent, pro-inflammatory cytokine with great potential for cancer immunotherapy. Several IL12 immunocytokines, based on tumor-targeted IgGs (13–15) or Ab fragments (16, 17), are in early-stage clinical trials against solid tumors (15, 18). Our group previously developed heterodimeric Ig Fc-fused IL12, termed mono-IL12-Fc, carrying one molecule of IL12 in the monovalent and naturally occurring heterodimeric form (19). Mono-mIL12-Fc exhibited much stronger antitumor potency with murine IL12 (mIL12) in murine solid tumor models by augmenting CD8^+^ T cell immune responses compared with those of wild-type Fc-based bivalent binding mIL12-Fc (bi-mIL12-Fc) carrying two molecules of mIL12. The human IL12 version of mono-IL12-Fc recently entered phase-I/II clinical trials (NCT04423029) for the treatment of solid tumors in combination with nivolumab (3, 20). However, mono-IL12-Fc lacks tumor-targeting ability.

To overcome this limitation, we used trastuzumab [brand name Herceptin (HCT)], which is a monoclonal Ab specific to the tumor-associated antigen human epidermal growth factor receptor 2 (HER2; also known as Neu or ERBB2) (21) to establish a series of human IgG1/4 hybrid heterodimeric Fc-based IgG-mono-mIL12 (hereafter termed HCT-mono-mIL12) variants carrying one molecule of mIL12, which vary in their binding affinities (*K*_D_) mainly through different dissociation rates (*k*_off_) for the tumor antigen HER2. We then investigated how the tumor antigen-binding kinetics and affinity of HCT-mono-mIL12 immunocytokines affect the localization and intratumoral penetration in solid tumors and thus the antitumor activity through activations of effector T cells in immunocompetent mice bearing syngeneic colon tumors. Our results demonstrate the profound impact of tumor antigen-binding kinetics on the antitumor efficacy of immunocytokines.

## Materials and methods

### Construction of HCT-mono-mIL12 variants

To design anti-HER2 HCT variants, we modeled a tertiary structure of an HCT-derivative bD2 Ab complexed with HER2 (22) based on the reported HCT–HER2 complex structure (PDB ID: 1N8Z) (21) using the online modeling server ABodyBuilder (23). The four residues R30, N30a, F53, and Y92 on complementarity-determining regions (CDRs) of the variable light chain (VL) of bD2 were substituted with Ala, either individually or jointly, to generate three HCT variants: HCT/46 with N30aA, HCT/130 with R30A and Y92A, and HCT/217 with F53A mutations. DNAs encoding the variable heavy chain (VH) and VL of HCT and its variants were subcloned in-frame into EW-RVT heterodimeric Fc-based HC plasmids pcDNA3.4-VH-EW HC(γ1/4)_CH3A_ [CH1 (γ1)–hinge (γ1)–CH2 (γ4)–CH3A (γ4, K360E/K409W)] and pcDNA3.4-VH-RVT HC(γ1/4)_CH3B_ [CH1 (γ1)–hinge (γ1)–CH2 (γ4)–CH3B (γ4, Q347R/D399V/F405T)], and the LC plasmid pcDNA3.4-VL-Cκ, respectively (19, 24). For the generation of heterodimeric Fc-based HCT-mono-mIL12, p40 with a 15-residue (G_4_S)_3_ linker and p35 with a 30-residue linker (L30, GGSSGSGSGSTGTSSSGTGTSAGTTGTSAS) were fused to the C-terminus of EW HC(γ1/4)_CH3A_ and RVT HC(γ1/4)_CH3B_, respectively. All constructs were confirmed by sequencing (Macrogen, Korea).

### Expression and purification of HCT-mono-mIL12 variants and other proteins

To produce HCT-mono-mIL12, three plasmids encoding the two different HC variants [VH-EW HC(γ1/4)_CH3A_-p40 and VH-RVT HC(γ1/4)_CH3B_-p35] and one of three LC variants for HCT/0.5 (HCT/46, HCT/130, or HCT/217) were transiently co-transfected at an equivalent molar ratio into cultured HEK293F cells in FreeStyle 293F medium (Invitrogen) following the standard protocol (19, 25). HCT-mono-mIL12 was purified from the culture supernatants after 6 to 7 d on a protein A–agarose chromatographic column (GE Healthcare) and extensively dialyzed to switch the solution to Dulbecco’s phosphate-buffered saline (PBS) buffer (2.67 mM KCl, 1.47 mM (KH_2_PO_4_), 137 mM NaCl, 8.1 mM Na_2_HPO_4_, pH 7.4). The purified proteins were further purified by fast protein liquid chromatography using a Superdex 200 10/300 column with an NGC Quest 10 chromatography system (Bio-Rad) (19) and finally formulated with PBS buffer. Unmodified HCT Ab and EW-RVT Fc proteins were purified as previously described (24, 26) and formulated with PBS buffer. Before cell treatment, the purified proteins were sterilized using a cellulose acetate membrane filter (0.22 μm; Corning) and Mustang Q membrane filter (0.8 μm; Pall, MSTG25Q6). Protein concentration was determined with the bicinchoninic acid kit (Thermo Fisher Scientific) and by measuring absorbance at 280 nm using the molar extinction coefficient calculated from the primary sequence (25). To determine the size and assembly pattern, size-exclusion chromatography (SEC) analysis of purified proteins was performed on the Agilent 1100 high-performance liquid chromatography system with a Superdex 200 10/300 GC column (10 mm × 300 mm, GE Healthcare) (19, 27). In SEC analysis, molecular mass standard markers, apoferritin (443 kDa), β-amylase (200 kDa), and alcohol dehydrogenase (150 kDa) from Sigma-Aldrich were used.

### Binding kinetics of HCT-mono-mIL12

Binding kinetics and affinity for the interactions of HCT-mono-mIL12 with HER2 antigen (Sino Biological Inc.) were measured using an Octet QKe instrument (ForteBio), as described previously (28). All kinetic experiments were conducted at 25°C with orbital shaking at 1000 rpm in a volume of 200 μL in 96-well black flat-bottom plates (VWR International, 82050-784). Each purified HCT and HCT-mono-mIL12 variant was diluted to 5 μg/mL in kinetics buffer [phosphate-buffered saline (PBS), pH 7.4, containing 0.02% (v/v) Tween 20] and directly immobilized onto anti-human IgG Fc capture biosensors (ForteBio) with an approximate 1.0 nm response. After an equilibration step of 180 s, the binding isotherms were monitored by exposing separate sensors simultaneously to different concentrations of HER2 antigen. The association of the antigen was measured for 100 s, followed by a dissociation step for 300 s. The association (*k*_on_) and dissociation rate (*k*_off_) constants as well as the equilibrium dissociation constant (*K*_D_) were determined by fitting to sensorgrams *via* the 1:1 binding model with a correlation coefficient (*R* ^2^) value equal to or greater than 0.99 in Octet Data Analysis software version 11.0 (ForteBio).

### Cell cultures

The mouse colon carcinoma CT26 and CT26-HER2/neu (human HER2/neu-expressing CT26 cells) cell lines (19, 29) were authenticated by DNA short-tandem repeat profiling (ABION CRO, Seoul, Korea) and used within 20 passages. The cell lines were maintained in Dulbecco’s modified Eagle medium containing 10% heat-inactivated fetal bovine serum (FBS; HyClone, Logan, UT, USA) and 1% penicillin-streptomycin (WelGENE, Daegu, Korea). The CT26-HER2/neu cell line was maintained by adding 0.68 mg/mL G418 antibiotic (Sigma-Aldrich) in the culture medium. Cell lines were routinely screened for *Mycoplasma* contamination (CellSafe, Gyeonggi-do, Korea).

### Preparation and activation of human peripheral blood mononuclear cells

Peripheral blood mononuclear cells (PBMCs) from healthy donors were acquired using protocols approved by the Institutional Review Board of Ajou University (approval ID: 201602-HM-001-01) (25). All donors provided written informed consent before blood collection into a BD Vacutainer (BD Biosciences, 367874). PBMCs were isolated using Ficoll-Paque Plus (GE Healthcare, 17-5442-03) density-gradient centrifugation (25). For long-term storage, PBMCs were resuspended with 10% dimethyl sulfoxide in FBS and stored in liquid nitrogen at 1–5 × 10^6^ cells/mL (28). For activation of PBMCs, the monocytes were partially depleted by plastic adherence, and non-adherent cells were resuspended at 1 × 10^6^ cells/mL in supplemented medium [RPMI 1640 plus 5% human AB serum (Irvine Scientific, Santa Ana, CA, USA), 10 mM HEPES, 0.006% (w/v) l-arginine monohydride, and 0.1% (w/v) dextrose] containing 10 μg/mL phytohemagglutinin (PHA; Sigma-Aldrich) and 20 IU/mL recombinant human IL2 (Thermo Fisher Scientific) and cultured for 3 d (28). The IL12R binding assay of Fc or HCT-mono-mIL12 for resting and activated PBMCs and the PBMC proliferation assay by stimulation with Fc or HCT-mono-mIL12 were performed as described previously (19).

### Mice

Four to five-week-old female BALB/c mice were purchased from Orient Bio (Seongnam, Korea) and tumor inoculation was performed when reaching 5–6 weeks of age. Mice were maintained according to the guidelines of the Institutional Animal Care and Use Committee of Ajou University. All animal studies were approved by the Institutional Animal Care and Use Committee (approval ID: 2017-0011 and 2020-0010) and conducted in accordance with the guidelines of the Animal and Ethics Review Committee of Ajou University (19, 29).

### Tumor inoculation and treatment

To establish the dual-flank tumor model, BALB/c mice were subcutaneously (s.c.) injected with CT26 cells (1 × 10^6^ cells in 100 μL PBS) into the left flank and with CT26-HER2/neu cells (1 × 10^6^ cells in 100 μL PBS) into the right flank. For induction of single-flank CT26-HER2/neu tumors, CT26-HER2/neu cells (1 × 10^6^ cells in 100 μL PBS) were s.c. injected into the flanks of BALB/c mice. When the mean tumor volume reached approximately 300 mm^3^ (after ~14 d of inoculation), the mice were randomized into treatment groups, and the corresponding HCT-mono-mIL12, HCT, Fc, or vehicle control group was intraperitoneally (i.p.) injected twice a week with an equivalent molar amount of 0.5 μg recombinant mIL12 (rmIL12) per mouse, as specified in the figure legend. Tumor volume (*V*) was evaluated using digital calipers and calculated by the formula *V* = *L* × *W* ^2^/2, where *L* and *W* are the long and short lengths of the tumor, respectively (25). The mice were euthanized with CO_2_ asphyxiation and some tumors were harvested.

### *In vivo* biodistribution analysis

Each HCT-mono-mIL12 was fluorescently labeled by conjugating with DyLight 680 and purified using DyLight 680 Antibody Labeling Kit (Thermo Fisher Scientific, 53056) in accordance with the manufacturer’s specifications (27). DyLight 680-labeled HCT-mono-mIL12 was then i.p. injected into BALB/c mice bearing dual-flank tumors of ~300 mm^3^ (n = 7 per group). Before imaging, the mice were anesthetized with 1.5–2.5% isoflurane (Piramal Critical Care) (27). The whole-body distribution profiles of the protein were determined *via in vivo* fluorescence using the IVIS imaging system (VISQUE *In vivo* Smart) at the indicated time points. After the final scan, tumor tissues and normal organs were excised and imaged *ex vivo*. Images were analyzed with an excitation wavelength of 683 nm and an emission wavelength of 703 nm. The mean radiant efficiency in the region of interest was quantified by radiant efficiency [photons/(s·cm^2^·steradian) per μW/cm^2^] using CleVue software (27).

### Flow cytometry

To determine the binding activity of HCT-mono-mIL12 for HER2-expressing CT26-HER2/neu cells, the cells were incubated with each protein at the indicated concentrations for 30 min, washed with PBS, and stained with anti-human IgG4-fluorescent isothiocyanate (FITC) (Sigma-Aldrich) for 30 min. For analysis of the numbers and phenotypes of CD4^+^ and CD8^+^ tumor-infiltrating lymphocytes (TILs), tumors were excised, mechanically dissociated into single-cell suspensions, and processed through a 70-μm wire-mesh screen (19, 25). Red blood cells were lysed with RBC Lysis Buffer (Thermo Fisher Scientific). For flow cytometry analysis, Abs specific for CD45 (clone 30-F11), CD3ϵ (clone 145-2C11 for mouse), CD8α (clone 53-6.7), CD4 (clone GK1.5), granzyme B (clone NGZB), IFNγ (clone XMG1.2), IL2 (clone JES6-5H4), TNFα (clone TN3-19.12), Ki-67 (clone solA15), and the isotype control Abs (rat IgG1, rat IgG2a, and rat IgG2b) from Thermo Fisher Scientific were used (29). Surface staining was performed at 4°C with Abs resuspended in PBS supplemented with 2% FBS and 2 mM ethylenediaminetetraacetic acid. For intracellular cytokine staining, cells were activated with phorbol 12-myristate 13-acetate (100 ng/mL) plus ionomycin (500 ng/mL) in a humidified incubator with 5% CO_2_ at 37°C for 16 h. Brefeldin A (BD Biosciences, 00-4506-51) was added for the last 6 h of culture to prevent protein transport from the endoplasmic reticulum to the Golgi apparatus. All intracellular staining was performed using a BD cytofix/cytoperm kit (BD Biosciences, 554714). After washing, the cells were analyzed by FACSCalibur (BD Biosciences) and flow cytometric data were analyzed with FlowJo ver10 software (Tree Star).

### Immunofluorescence microscopy of tumor tissues

Excised tumor tissues from BALB/c tumor-bearing mice (TBM) were processed to analyze the distribution and diffusion of HCT-mono-mIL12 and CD4^+^ and CD8^+^ TILs using established protocols (25, 27). Briefly, the excised tumors were fixed in 4% paraformaldehyde overnight at 4°C, cryo-protected in 30% (w/v) sucrose overnight at 4°C, and then frozen in OCT (Tissue-Tek) embedding medium. For immunofluorescence (IF) staining, cryosections were cut at 10 μm thickness and blocked with 2% bovine serum albumin in PBST [PBS, pH 7.4, 0.01% (v/v) Tween-20] for 1 h at 25°C. HCT-mono-mIL12 was detected with FITC-conjugated mouse anti-human IgG4 Ab (1:50; Sigma-Aldrich, F9890) for 1.5 h at 25°C. After another blocking step for 1 h, the slides were incubated with rat anti-mouse CD31 monoclonal Ab (mAb; 1:50; BD Pharmingen, 550274) for 4 h at 25°C. Next, the slides were washed and further incubated with TRITC-conjugated goat anti-rat Abs (1:50; Millipore, AP136R) for 1.5 h at 25°C. For staining of CD4^+^ and CD8^+^ TILs, tissue sections were blocked with 5% normal goat serum in PBST for 1 h at 25°C and treated with rabbit anti-mouse CD8α mAb (1:100; Abcam, ab217344) or rabbit anti-mouse CD4 mAb (1:100; Abcam, ab183685) for 4 h at 25°C. The primary Ab was detected with Alexa Fluor 488-conjugated goat anti-rabbit Ab (1:100; Thermo Fisher Scientific, A-11008). Rat anti-IFNγ mAb (1:100; Invitrogen, MM700) was used for staining of IFNγ-positive TILs. Cell nuclei were stained with Hoechst 33342 in 0.01% PBST for 10 min at 25°C. After mounting the glass slides with Fluorescence Mounting Medium (Dako), center-focused single–z-section images were recorded on a Zeiss LSM 710 system and analyzed with ZEN software (Carl Zeiss).

### Analysis of IF images

For IF images acquired from each tissue sample, the fluorescence intensity and number of positively stained cells were quantified using ImageJ software and presented as the relative staining intensity (%) compared to the corresponding control. Distribution of HCT-mono-mIL12 according to distance from the blood vessels was quantified by measuring the fluorescence intensity (30). In detail, images were converted to 8-bit black-and-white binary images, and the images displaying anti-CD31 staining were overlayed with the corresponding field of view displaying HCT-mono-mIL12 staining. After setting the distance from the blood vessels up to ~150 μm, the fluorescence intensities were automatically measured every 1 μm with an intensity setting of 255 (white, background) and 0 to 254 (black, HCT-mono-mIL12). For each HCT-mono-mIL12, 2–3 blood vessels were randomly selected per field and the fluorescence intensity according to the distance from the blood vessels was measured twice for each vessel in a random direction, resulting in 4–6 measurements per field (9).

To quantify the number of CD4-, CD8-, and IFNγ-positive T cells in the tumor sections, each image was converted to 8-bit black-and-white binary images and cell counting was performed by setting a threshold and relating it to the number of cells per millimeter squared (mm^2^) (31).

### Statistical analysis

Data are presented as representative images for imaging experiments, mean ± SEM for pooled data, or mean ± SD for representative assays involving at least three independent experiments, unless specified otherwise. Differences between experimental groups and controls were analyzed for statistical significance by unpaired two-tailed Student’s *t*-tests. One-way analysis of variance with the Newman–Keuls multiple-comparison *post hoc* test was performed to determine the significance of *in vivo* tumor growth data using GraphPad Prism software (GraphPad, Inc.). No corrections were implemented in the statistical tests. A p value < 0.05 was considered to denote statistical significance.

## Results

### Design and characterization of HCT-mono-mIL12 variants with different anti-HER2 binding kinetics and affinities

**Figure 1:**
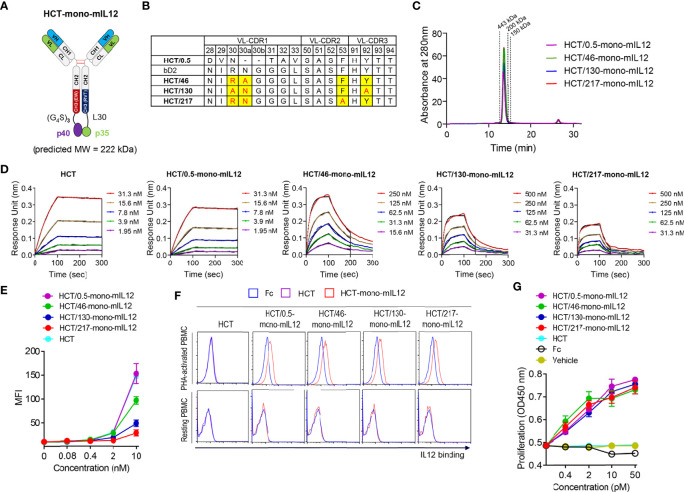
Generation and characterization of HCT-mono-mIL12 variants with different anti-HER2 binding kinetics. **(A)** Schematic diagram of HCT-mono-mIL12, where the two subunits of mIL12, p40 and p35, were separately fused to the C-terminus of heterodimeric Fc-based HC *via* (G_4_S)_3_ and a 30-residue linker (L30), respectively. **(B)** The sequence alignment of VL-CDRs of HCT variants highlighting the mutated residues in VL-CDRs of bD2. **(C)** SEC elution profiles of the purified HCT-mono-mIL12 (30 μg loading amount each). The dotted lines indicate the elution positions of molecular mass standard markers: 443 kDa (apoferritin), 200 kDa (β-amylase), and 150 kDa (alcohol dehydrogenase). **(D)** Representative binding isotherms of the immobilized HCT or HCT-mono-mIL12 to soluble HER2 antigen, measured by bio‐layer interferometry. The concentrations of HER2 analyzed are indicated in different colors as defined in the in-graph legends. The binding kinetics (*k*_on_ and *k*_off_) and affinities (*K*_D_), shown in **Table 1**, were obtained by global fitting of the association and dissociation phases (indicated by the black vertical line) with *R* ^2^ ≥ 0.99. **(E)** Dose-dependent binding of HCT (control) or HCT-mono-mIL12 to CT26-HER2/neu cells, measured by flow cytometry and represented by the mean fluorescence intensity (MFI). **(F)** Binding activities of Fc (40 nM), HCT (40 nM), or HCT-mono-mIL12 variants (40 nM) for resting and PHA-activated PBMCs, analyzed by flow cytometry. **(G)** Proliferation of PHA-activated PBMCs after 72 h culture with the indicated concentrations of Fc (control), HCT (control), or HCT-mono-mIL12 variants. Data in **(C, D, F)** are representative of three independent experiments. Data in **(E, G)** represent the mean ± SD (n=3).

Given that human IL12 does not cross-react with murine IL12R (19), mIL12 was employed for the *in vivo* study in mice. To generate HCT-mono-mIL12, we separately fused the two subunits (p35 and p40) of mIL12 to the C-terminus of each heterodimeric Fc heavy chain of HCT through a respectively different linker (**Figure 1A**). The heavy chain of HCT-mono-mIL12 was a human IgG1/4 hybrid with an EW-RVT heterodimeric Fc mutation pair (24) to minimize the binding to Fcγ receptors on immune cells and thus avoid depletion by Fcγ-expressing cells (32). The binding affinity of the purified HCT-mono-mIL12 for HER2 antigen was in the sub-nanomolar range of the equilibrium dissociation constant (*K*_D_) ≈ 0.54 nM, as determined by bio‐layer interferometry (**Table 1**). We named the wild-type HCT-based IgG-mono-mIL12 as HCT/0.5-mono-mIL12 to indicate the HER2 binding affinity.

**Table 1:** Binding kinetics of interactions between soluble HER2 and immobilized HCT-mono-mIL12, as measured by bio-layer interferometry.

	Binding kinetic parameters						
	*k*_on_ (M^-1^s^-1^, ×10^5^)	*k*_off_ (s^-1^, ×10^-3^)	*K*_D_ (nM)	*R* ^2(a)^	*k*_on_ ratio	*k*_off_ ratio	*K*_D_ ratio
					(variant *vs*. HCT/0.5-mono-mIL12)^(b)^		
HCT	3.63 ± 0.10	0.13 ± 0.02	0.36 ± 0.06	0.99	–	–	–
HCT/0.5-mono-mIL12	3.27 ± 0.11	0.18 ± 0.02	0.54 ± 0.08	0.99	1	1	1
HCT/46-mono-mIL12	2.14 ± 0.06	9.85 ± 0.13	46.1 ± 1.46	0.99	0.65	55	85
HCT/130-mono-mIL12	1.45 ± 0.06	18.90 ± 0.54	130.0 ± 6.48	0.99	0.54	105	241
HCT/217-mono-mIL12	1.34 ± 0.08	29.20 ± 0.67	217.0 ± 13.90	0.99	0.46	162	402

^(a)^Correlation coefficient value for the fitting.

^(b)^The ratio was obtained versus the corresponding value of HCT/0.5-mono-mIL12.

To construct a series of HCT variants with reduced affinity for HER2, we mutated HER2-binding paratope residues in the VL-CDRs of bD2 (22) by structure-guided site-directed mutagenesis based on the reported crystal structure of the HCT-HER2 complex (21) (**Figure 1B**; **Supplementary Figure 1A**). Using the resulting HCT affinity variants, we created three HCT-mono-mIL12 variants, named HCT/46-, HCT/130-, and HCT/217-mono-mIL12, with *K*_D_ ≈ 46, 130, and 217 nM for HER2 antigen, respectively (**Table 1**). All variants were purified in the correctly assembled form (molecular weight ≈ 222 kDa) with high purity (≥ 95%), as determined by sodium dodecyl sulfate-polyacrylamide gel electrophoresis (**Supplementary Figure 1B**) and SEC analysis (**Figure 1C**). The main contributor to the reduced affinity of the variants was a 55−162-fold increase in the dissociation rate constant (*k*_off_) rather than the smaller ~2-fold decrease in the association rate constant (*k*_on_) compared with those of the parental HCT/0.5-mono-mIL12 (**Figure 1D** and **Table 1**). HCT-mono-mIL12 variants exhibited dose-dependent binding activity for HER2 expressed on the surface of CT26-HER2/neu cells (**Figure 1E**), with the binding strength profiles matching the binding affinity for soluble HER2 antigen. HCT/0.5-mono-mIL12 showed equivalent binding activity for HER2 to that of unmodified HCT (**Figure 1E**), indicating that the C-terminal fusion of mIL12 does not compromise the HER2 binding activity of HCT/0.5-mono-mIL12.

Human PBMCs stimulated with the T cell mitogen PHA were utilized to evaluate the IL12R-binding activities of HCT-mono-mIL12 variants, since mIL12 cross-reacts with human IL12R on activated human T cells (19). Compared with the Fc and HCT controls, all variants bound to PHA-activated PBMCs but not to unstimulated PBMCs (**Figure 1F**), demonstrating the IL12R binding specificity. Moreover, all variants induced the proliferation of PHA-activated PBMCs in a dose-dependent manner, showing almost the same efficacy at the equivalent concentration (**Figure 1G**). Thus, HCT/0.5-mono-mIL12 and its variants manifested indistinguishable activities for IL12R binding and stimulation, despite exhibiting different binding parameters for HER2.

### Tumor targeting and retention of HCT-mono-mIL12 requires anti-HER2 affinity greater than a threshold

**Figure 2:**
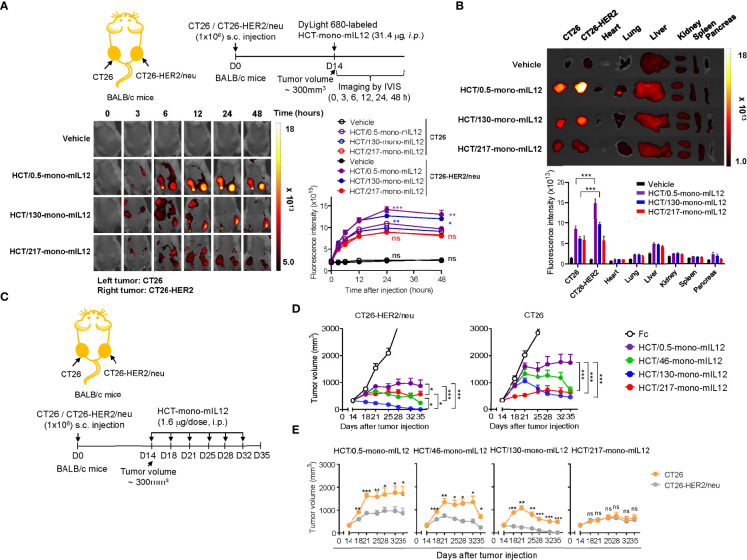
Selective tumor accumulation and antitumor activity of HCT-mono-mIL12 requires anti-HER2 affinity above a certain threshold. **(A)** Representative whole-body fluorescence images showing the biodistribution of DyLight 680-labeled HCT-mono-mIL12 according to the indicated time after a single i.p. injection of 31.4 μg into BALB/c mice bearing dual-flank tumors of CT26 (left flank) and CT26-HER2/neu (right flank) cells assessed at a tumor volume of ~300 mm^3^; additional images are shown in **Supplementary Figure 2A**. *Right*, fluorescence intensities in CT26 and CT26-HER2/neu tumor tissues, quantified by radiant efficiency. Data represent means ± SEM (n = 7). *p < 0.05, **p < 0.01, ***p < 0.001 between CT26 and CT26-HER2/neu tumors; ns, not significant. **(B)** *Ex vivo* analysis of fluorescence intensity of the excised tumors and normal organs 48 h after a single i.p. injection of DyLight 680-labeled HCT-mono-mIL12, as shown in **(A)**. Tumor tissue and normal organs of one representative mouse from each group are shown; additional images are shown in **Supplementary Figure 2B**. *Lower*, fluorescence intensities of tumors and organs quantified by radiant efficiency. In **(A, B)**, vehicle indicates PBS buffer as control. Data represent means ± SEM (n = 7). *** p < 0.001 between the indicated groups. **(C–E)** Treatment scheme of BALB/c mice bearing dual-flank tumors of CT26 (left flank) and CT26-HER2/neu (right flank) cells initiated at a tumor volume of ~300 mm^3^ with i.p. injection of HCT-mono-mIL12 at 1.6 μg per dose (an equimolar amount of 0.5 μg rmIL12 per dose) twice weekly **(C)** to determine the antitumor efficacy **(D, E)**. In **(D, E)**, the growth of CT26 and CT26-HER2/neu tumors, measured by tumor volume, is shown separately **(D)** or comparatively **(E)** according to HCT-mono-mIL12. Data represent means ± SEM (n = 12–14 per group). *p < 0.05, **p < 0.05, ***p < 0.001 between the indicated groups **(D)** and between CT26 and CT26-HER2/neu tumors; ns, not significant **(E)**. Data are pooled from two independent experiments with at least four mice per group.

To determine the *in vivo* tumor-targeting specificity of HCT-mono-mIL12, we first evaluated the biodistribution of DyLight 680-labeled HCT-mono-mIL12 in BALB/c mice bearing dual-flank syngeneic tumors (HER2-expressing CT26-HER2/neu tumors in the right flank and HER2-negative CT26 tumors in the left flank) at an average tumor volume of approximately 300 mm^3^ (**Figure 2A**). To obtain detectable whole-body imaging, a relatively high dose of 31.4 μg HCT-mono-mIL12 (corresponding to the molar amount of 10 μg rmIL12) was i.p. injected and then a temporal biodistribution was obtained up to 48 h. During the experiments, we did not observe any noticeable systemic toxicities such as body weight loss and death. Both HCT/0.5-mono-mIL12 (*K*_D_ ≈ 0.54 nM) and HCT/130-mono-mIL12 (*K*_D_ ≈ 130 nM) manifested preferential accumulation in CT26-HER2/neu tumors over CT26 tumors, with a maximum peak at 24 h after injection and then began to decline, as compared to the distribution in normal tissues (**Figures 2A, B**; **Supplementary Figure 2**), demonstrating their *in vivo* targeted selectivity for HER2-expressing tumors. Conversely, HCT/217-mono-mIL12 with the lowest HER2 affinity (*K*_D_ ≈ 217 nM) failed to show preferential accumulation in CT25-HER2/neu tumors over 2 days as compared to that in CT26 tumors (**Figures 2A, B**; **Supplementary Figure 2**). These results demonstrated that an anti-HER2 affinity higher than a certain threshold (*K*_D_ ≈ 130 nM) is required; thus, the affinity of *K*_D_ ≈ 217 nM is too low for selective tumor targeting and retention of HCT-mono-mIL12 in CT25-HER2/neu tumors.

### HCT-mono-mIL12 shows more potent *in vivo* antitumor activity for HER2-positive tumors than HER2-negative tumors

Immunocompetent BALB/c mice induce adaptive immune responses against human HER2/neu antigen (19). To assess the advantage of the HER2-expressing tumor-targeting selectivity property of HCT-mono-mIL12, we treated BALB/c mice bearing the dual-flank tumors with HCT-mono-mIL12, once reaching an average tumor volume of approximately 300 mm^3^, *via* i.p. injection at 1.6 μg per dose (approximately 80 μg/kg per dose; an equimolar amount of 0.5 μg rmIL12 per dose) twice a week for a total of six doses (**Figure 2C**). Compared with that of the Fc-treated control, all HCT-mono-mIL12 variants showed markedly slower and/or regressed growth of both CT26 and CT26-HER2/neu tumors (**Figures 2D, E**; **Supplementary Figure 3**). However, with the exception of HCT/217-mono-mIL12, which exhibited equipotent growth inhibitory activity for both CT26-HER2/neu and CT26 tumors without selectivity, the other HCT-mono-mIL12 variants with anti-HER2 affinity higher than 217 nM elicited much stronger antitumor activity for CT26-HER2/neu tumors than for CT26 tumors, demonstrating the more potent antitumor activity for targeted tumors than non-targeted tumors. These variants displayed antitumor activity in the order of HCT/130-mono-mIL12 (*K*_D_ ≈ 130 nM) > HCT/46-mono-mIL12 (*K*_D_ ≈ 46 nM) > HCT/0.5-mono-mIL12 (*K*_D_ ≈ 0.54 nM) (**Figures 2D, E**), demonstrating an inverse relationship between anti-HER2 affinity and antitumor potency. The most potent HCT/130-mono-mIL12 variant could fully control the growth of CT26-HER2/neu tumors, resulting in complete tumor eradication at the end of the treatment period (**Supplementary Figure 3**). By contrast, HCT/0.5-mono-mIL12 with the highest affinity for HER2 showed the weakest antitumor activity, with a 0% (n = 0/14) rate of tumor-free survival in the mice (**Supplementary Figure 3**). These results demonstrated the beneficial antitumor activity of HCT-mono-mIL12 against HER2-expressing tumors over HER2-negative tumors and the importance of HER2 binding kinetics in determining the antitumor activity. The antitumor activity of HCT-mono-mIL12 observed against the non-target CT26 tumor on the opposite flank could be explained by systemic antitumor immune responses (19) and/or by non-specific local accumulation in the tumor, representing the so-called enhanced permeability and retention effect (33), as observed in the biodistribution analysis (**Figures 2A, B**).

### *In vivo* antitumor potency of HCT-mono-mIL12 depends on the anti-HER2 binding kinetics

**Figure 3:**
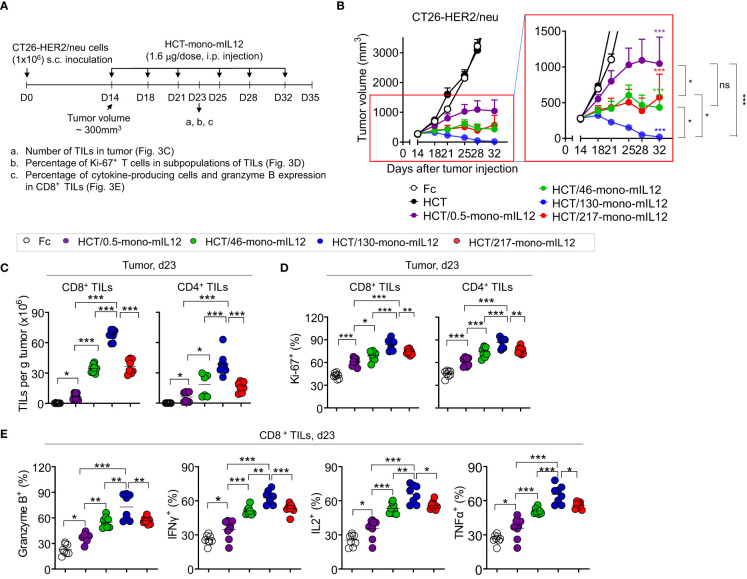
*In vivo* antitumor efficacy of HCT-mono-mIL12 varies according to its anti-HER2 binding kinetics. **(A–E)** Treatment scheme of BALB/c mice bearing a single-flank tumor of CT26-HER2/neu cells initiated at a tumor volume of ~300 mm^3^ with i.p. injection of HCT-mono-mIL12 at 1.6 μg per dose (an equimolar amount of 0.5 μg rmIL12 per dose) twice weekly **(A)** to determine the antitumor activity **(B)** and its effect on the number and function of CD4^+^ and CD8^+^ TILs **(C–E)** assessed on day 23 after tumor inoculation [see a–c of **(A)**]. In **(A)**, the arrows indicate each time point for the treatment or assay. In **(B)**, data represent means ± SEM (n = 14 per group). The region boxed in red is enlarged to the right to better visualize the differences in the antitumor activity among HCT-mono-mIL12 variants. **(C–E)** Number of CD4^+^ and CD8^+^ TILs **(C)** and percentage of Ki-67-expressing **(D)** and cytokine-producing or granzyme B-expressing cells **(E)** among CD4^+^ and/or CD8^+^ TILs in the CT26-HER2/neu tumor-bearing mice analyzed by flow cytometry. In **(C–E)**, each symbol represents the value obtained from individual mice (n ≥ 8 per group), and midlines represent the means of two pooled experiments. In **(B–E)**, *p < 0.05, **p < 0.01, ***p < 0.001 between the indicated groups as determined by one-way analysis of variance with the Newman-Keuls *post-hoc* test; ns, not significant. Data are pooled from two independent experiments with at least four mice per group.

To further evaluate the relationship between HER2 binding kinetics and the antitumor potency of HCT-mono-mIL12, we repeated the *in vivo* study in BALB/c mice bearing a single-flank tumor of CT26-HER2/neu cells (**Figure 3A**). Compared with those of the Fc and unmodified HCT controls, HCT-mono-mIL12 variants resulted in significant growth suppression and/or regression of established CT26-HER2/neu tumors (**Figure 3B**). Notably, HCT/130-mono-mIL12, with ~241-fold lower affinity for HER2 due to a ~105-fold faster dissociation rate from HER2 compared with that of HCT/0.5-mono-mIL12, induced the most robust tumor regression, resulting in a 79% (n = 11/14) rate of tumor-free survival in the mice, which was much higher than the rates of 57% (n = 8/14) for HCT/217-mono-mIL12, 29% (n = 4/14) for HCT/46-mono-mIL12, and 0% (n = 0/14) for HCT/0.5-mono-mIL12 (**Supplementary Figure 4**). Accordingly, the antitumor activity was ranked in the following order: HCT/130-mono-mIL12 > HCT/46-mono-mIL12 ≈ HCT/217-mono-mIL12 > HCT/0.5-mono-mIL12 (**Figure 3B**), in good agreement with that observed in the dual-flank tumor model. These results indicated an inverse relationship between the anti-HER2 binding kinetics and antitumor activity of HCT-mono-mIL12, except for HCT/217-mono-mIL12.

### HCT-mono-mIL12-induced tumor infiltration and intratumoral activation of effector T cells depend on anti-HER2 binding kinetics

To elucidate the cellular mechanisms underlying the potent antitumor activity of HCT-mono-mIL12, we analyzed the numbers and phenotypes of CD4^+^ and CD8^+^ TILs in tumors isolated from CT26-HER2/neu–TBM on day 23 after three treatments with HCT-mono-mIL12, because the six-dose administration of HCT/130-mono-mIL12 completely eradicated the tumors in the mice (**Figure 3B**). Compared with those of Fc-treated controls, treatment with HCT-mono-mIL12 variants resulted in significant increases in the populations of CD8^+^ and CD4^+^ TILs (**Figure 3C**) and their percentages expressing Ki-67, a proliferation marker, in tumor sites (**Figure 3D**). The HCT-mono-mIL12 variants significantly enhanced the frequencies of granzyme B-, IFNγ-, IL2-, and TNFα-expressing CD8^+^ TILs, with HCT/130-mono-mIL12 showing the greatest effect, as compared with those observed in the Fc-treated control, demonstrating their increased cytotoxic potential (**Figure 3E**). The relative magnitude of these effects correlated closely with the antitumor activity, with HCT/130-mono-mIL12 showing the strongest effects (**Figures 3C–E**), accounting for the strongest tumor control with this variant (**Figure 3B**). Together, these data suggest that the tumor infiltration and functionality of T cells in tumors, as the likely primary effector cells for HCT-mono-mIL12-mediated antitumor efficacy (19), are affected by the anti-HER2 binding kinetics.

### Intratumoral penetration of HCT-mono-mIL12 depends on anti-HER2 binding kinetics

**Figure 4:**
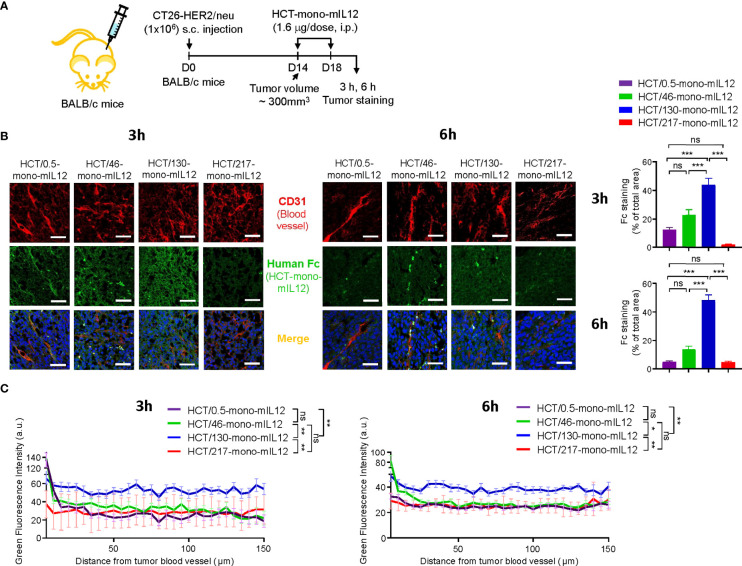
Intratumoral penetration of HCT-mono-mIL12 depends on its anti-HER2 binding kinetics. **(A–C)** Treatment scheme of CT26-HER2/neu–tumor-bearing mice initiated at a tumor volume of ~300 mm^3^ with i.p. injection of HCT-mono-mIL12 at 1.6 μg per dose (an equimolar amount of 0.5 μg rmIL12 per dose) two times, on days 14 and 18 after tumor inoculation **(A)**, to determine the intratumoral penetration of HCT-mono-mIL12 in tumor tissues excised from the mice at 3 h or 6 h after the second dosing on day 18 by IF staining **(B)** and for quantification of the fluorescence intensity from the nearest blood vessel in the tumor section **(C)**. In **(A)**, the arrows indicate each time point for treatment or assay. **(B)** Representative IF images depicting intratumoral diffusion of HCT-mono-mIL12 (human Fc staining with FITC, green) in relation to the blood vessels (CD31 staining with TRITC, red). Blue represents nuclei staining. Image magnification, ×200; scale bar, 50 μm. *Right*, quantification of positive areas of Fc staining (green) analyzed by ImageJ software. **(C)** Fluorescence intensity of HCT-mono-mIL12 in **(B)** quantified according to the distance from the nearest blood vessel in the tumor tissues. Each line represents the value of fluorescence intensity averaged every 5 μm. In **(B, C)** data represent mean ± SEM of four fields per tumor (n = 3 per group). *p < 0.05, **p < 0.01, ***p < 0.001 between the indicated groups; ns, not significant.

To elucidate the anti-HER2 binding kinetics-dependent antitumor activity of HCT-mono-mIL12, we next determined the paracellular penetration and spread within tumor tissues by IF staining for tumors excised from HCT-mono-mIL12-treated CT26-HER2/neu–TBM at 3 h and 6 h after the second dosing (**Figure 4A**). IF staining revealed that the highest-affinity variant HCT/0.5-mono-mIL12 was primarily detected in the vicinity of tumor blood vessels, whereas the lower-affinity variants HCT/46- and HCT/130-mono-mIL12 more deeply penetrated the tumor tissue far from the vessels, showing wider diffusion throughout vascularized tumors at both 3 h and 6 h after treatment over circulation time (**Figure 4B**). In particular, HCT/130-mono-mIL12 was evenly distributed throughout the tumor tissues with a ~3- and 7-fold wider area than found for HCT/0.5-mono-mIL12 at 3 h and 6 h, respectively (**Figure 4B**). IF staining intensities of all immunocytokines were stronger at 3 h than at 6 h (**Figure 4B**), indicating that they are gradually depleted in the tumor tissue over time.

We further analyzed the IF images to quantify each HCT-mono-mIL12 according to the distances from the nearest blood vessels. Compared with those of HCT/0.5-, HCT/46-, and HCT/217-mono-mIL12, which exhibited very similar distribution profiles, HCT/130-mono-mIL12 exhibited the highest levels across the proximal and distal regions, reaching up to 150 μm from the blood vessels, at both 3 h and 6 h (**Figure 4C**). Thus, HCT/130-mono-mIL12 manifested the greatest ability to diffuse into the solid tumor at distal regions from the blood vessels, underscoring the importance of anti-HER2 binding kinetics for maximal intratumoral diffusion. Although the lowest-affinity HCT/217-mono-mIL12 variant showed the poorest localization in the tumors (**Figure 4B**), consistent with its lowest biodistribution in tumors (**Figures 2A, B**), the intratumoral distribution of this variant in the distal regions from the blood vessels was similar to those of HCT/0.5- and HCT/46-mono-mIL12 (**Figure 4C**), explaining the detected antitumor activity.

### Intratumoral distribution of CD4^+^ and CD8^+^ TILs correlates with the intratumoral penetration of HCT-mono-mIL12

**Figure 5:**
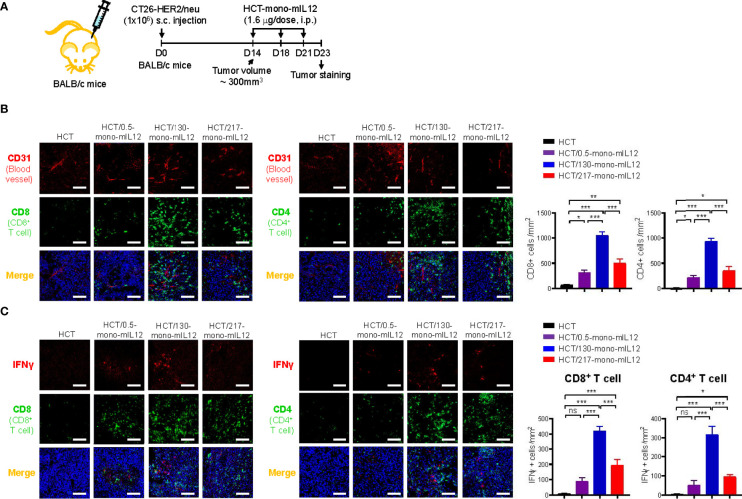
Intratumoral distribution of CD4^+^ and CD8^+^ TILs correlates strongly with the intratumoral penetration of HCT-mono-mIL12. **(A–C)** Treatment scheme of CT26-HER2/neu tumor-bearing mice initiated at a tumor volume of ~300 mm^3^ with i.p. injection of HCT-mono-mIL12 at 1.6 μg per dose (an equimolar amount of 0.5 μg rmIL12 per dose) three times on day 14, 18, and 21 after tumor inoculation **(A)** to determine the intratumoral distribution of total CD4^+^ and CD8^+^ TILs in relation to the blood vessels **(B)** and IFNγ-producing cells among CD4^+^ and CD8^+^ TILs **(C)** at 2 days after the third dosing determined by IF staining. In **(A)**, the arrows indicate each time point for treatment or assay. In **(B, C)**, tumor tissues were excised and stained for CD4 or CD8 (Alexa Fluor 488, green) with CD31 (TRITC, red) **(B)** and/or IFNγ (TRITC, red) **(C)**. Blue represents nuclei staining. Image magnification, ×200; scale bar, 50 μm. The bar graphs depict the number of the indicated cells per mm^2^ in a tumor section. Data represent mean ± SEM of four fields per tumor (n = 3 per group). *p < 0.05, **p < 0.01, ***p < 0.001 between the indicated groups; ns, not significant.

In addition to the tumor-homing property of effector T cells, their sufficient presence in deeper regions from the vessels is strongly correlated with better antitumor efficacy against solid tumors. Thus, we analyzed the distribution of CD4^+^ and CD8^+^ T cells in tumor tissues excised from CT26-HER2/neu–TBM on day 23 after three treatments with HCT-mono-mIL12 (**Figure 5A**). Treatment with unmodified HCT negligibly induced the tumor infiltration of CD4^+^ and CD8^+^ T cells, whereas HCT-mono-mIL12 treatment triggered tumor infiltration of those cells (**Figure 5B**). Both CD4^+^ and CD8^+^ T cells were located near the blood vessels for the tumors treated with the highest-affinity HCT/0.5-mono-mIL12 variant, but were dispersed more evenly and abundantly in the tumors treated with the lower-affinity variants, most prominently HCT/130-mono-mIL12 (**Figure 5B**). Further, when activated CD4^+^ and CD8^+^ T cells were detected by co-staining with IFNγ, the distribution tendency was similar to that of total CD4^+^ and CD8^+^ T cells in the tumors (**Figure 5C**), indicating that the TILs are activated to kill tumor cells in the TME. However, IFNγ staining was partially colocalized with CD8^+^ or CD4^+^ TILs, indicating that the activated CD8^+^ and CD4^+^ T cells secrete IFNγ with little intracellular accumulation (34). Quantitation of T cells (per mm^2^) revealed that the number of total and IFNγ-expressing CD4^+^ and CD8^+^ T cells in HCT/130-mono-mIL12-treated tumors increased by ~3-fold and ~6-fold, respectively, as compared to those in tumors treated with HCT/0.5-mono-mIL12 (**Figures 5B, C**), in line with the results of the flow cytometry analyses quantifying the numbers of total and activated CD4^+^ and CD8^+^ TILs (**Figures 3C–E**). Accordingly, tumor infiltration and intratumoral activation of CD4^+^ and CD8^+^ T cells in response to the mIL12 moiety correlated well with the intratumoral penetration of HCT-mono-mIL12 and the subsequent antitumor potency in the CT26-HER2/neu–TBM model, indicating that the greater tumor penetration of HCT-mono-mIL12 directly translates into the enhancement of therapeutic effects.

## Discussion

Immunocytokines, including IL12 immunocytokines, are promising agents for cancer immunotherapy by activating antitumor effector immune cells to kill tumor cells in the TME. However, the limited tumor localization, intratumoral penetration, and/or heterogeneous spread of immunocytokines in the TME, especially for solid tumors, lead to large untargeted regions that escape therapy. Based on the construction of HCT-mono-mIL12 variants with distinct anti-HER2 binding kinetics, we here provide the first *in vivo* evidence that tumor antigen binding kinetics and affinity play critical roles in tumor retention and intratumoral diffusion of the immunocytokine, thereby determining the tumor infiltration and intratumoral distribution of antitumor effector T cells and consequently their antitumor efficacy in immunocompetent TBM models. Our findings suggest that in the design of antitumor immunocytokines, the tumor antigen-binding kinetics and affinity should be adjusted to achieve optimal tumor retention and intratumoral diffusion, thereby effectively activating immune cells in the TME for potent antitumor efficacy.

**Figure 6:**
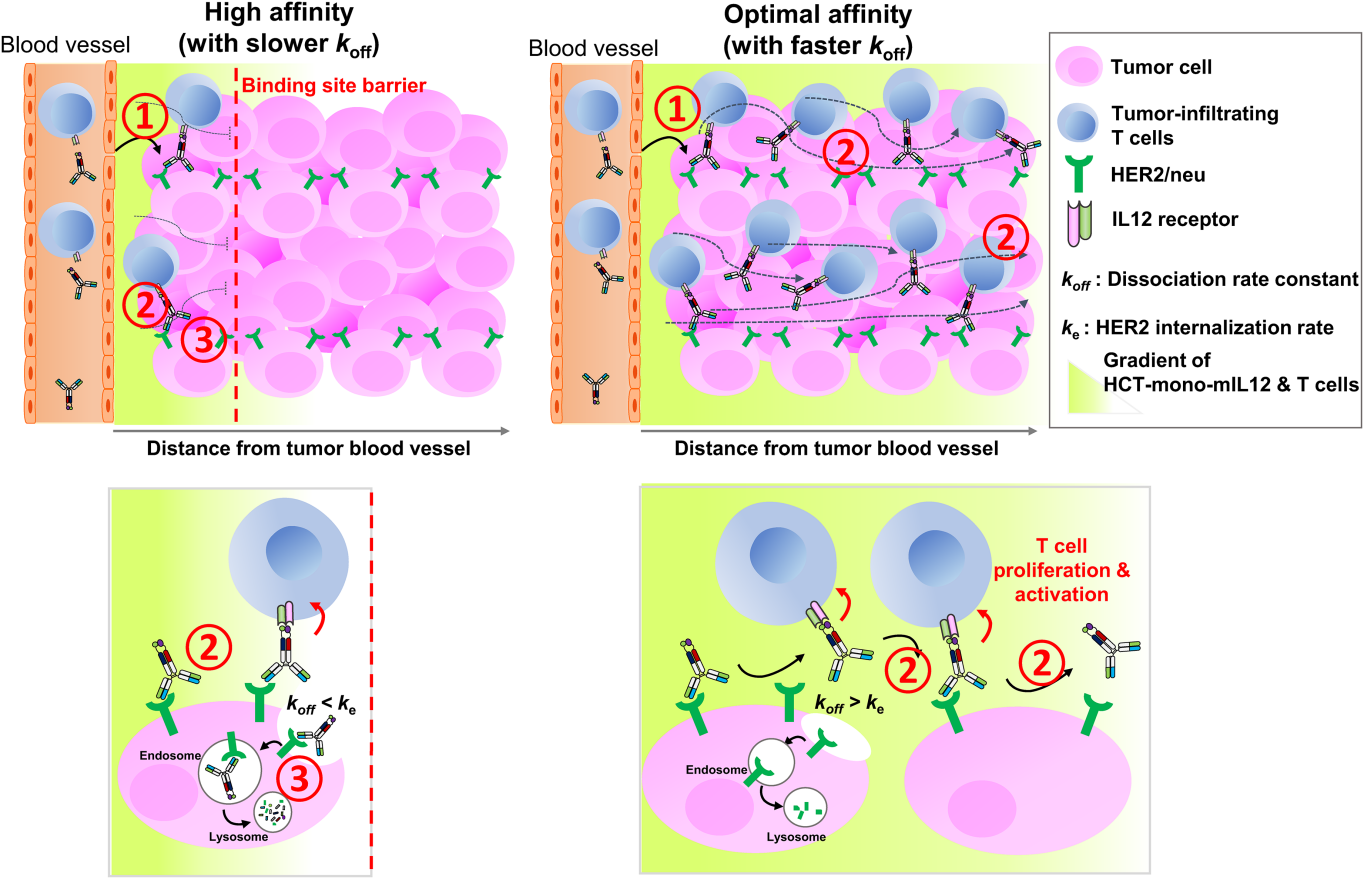
Schematics of *in vivo* intratumoral penetration of HCT-mono-mIL12 as a function of anti-HER2 binding kinetics, and its effect on tumor infiltration and activation of CD4^+^ and CD8^+^ T cells in the TME. The tumor localization and intratumoral penetration of HCT-mono-mIL12 are governed by three major steps: (1) vascular transport (extravasation) and tumor retention, (2) interstitial transport, and (3) HER2 receptor-mediated clearance. For tumor retention after extravasation (step 1), HCT-mono-mIL12 requires an anti-HER2 affinity above a threshold to strongly bind tumor cells; otherwise, it will be systemically eliminated. The interstitial transport (step 2) and HER2 receptor-mediated clearance (step 3) are mainly governed by the dissociation rate constant (*k*_off_) for HER2 antigen. In step 2, the slower the *k*_off_, the longer it takes to diffuse over a certain distance (*left panel*) and the faster the *k*_off_, the farther it transports across the tumor interstitium (*right panel*). In step 3, a slower *k*_off_ than the HER2 internalization rate (*k*_e_) leads to rapid depletion of HCT-mono-mIL12 (*left panel*) and a *k*_off_ faster than *k*_e_ leads to the extracellular presence and deeper penetration of HCT-mono-mIL12 (*right panel*). A deep and wide distribution of HCT-mono-mIL12 can induce the tumor infiltration of CD4^+^ and CD8^+^ TILs into the distal regions from the blood vessels and/or stimulate the *in situ* proliferation and activation of the TILs to kill tumor cells, resulting in profound antitumor efficacy.

The rate and extent of tumor accumulation and intratumoral penetration of systemically administered immunocytokines, including HCT-mono-mIL12, can be determined by three major steps (1): extravasation and tumor retention; (2) interstitial transport, representing intratumoral diffusion through the tumor tissue; and (3) local clearance by tumor antigen receptor-mediated internalization and degradation (**Figure 6**). Regarding the first step of extravasation and tumor retention, the molecular size and anti-HER2 affinity of HCT-mono-mIL12 were found to be crucial for HER2-expressing solid tumors. Since all of the HCT-mono-mIL12 variants have nearly the same size, their vascular transport from the blood vessels to tumors would be the same. However, HCT-mono-mIL12 needs to strongly bind to HER2 at a certain threshold level to achieve durable tumor retention (**Figure 6**, step 1); otherwise, it will be systemically eliminated through rapid diffusion out of the tumor. In the dual-flank tumor model, HCT/0.5- and HCT/130-mono-mIL12 selectively accumulated in HER2-expressing CT26-HER2/neu tumors over HER2-negative CT26 tumors, whereas HCT/217-mono-mIL12 could not, suggesting that tumor retention only occurs above a threshold of anti-HER2 affinity, below which unbound HCT-mono-mIL12 undergoes rapid systemic clearance through diffusion out of tumors due to the tumor’s high capillary permeability (6). Specifically, we found that a threshold affinity equal to or greater than 130 nM is required for tumor retention of HCT-mono-mIL12, in line with previous reports showing that a minimal affinity between 10^-7^ and 10^-8^ M is required for significant tumor localization of antitumor Abs in mouse tumor models (7, 8).

The next two steps, relating to the transport and local clearance of tumor-retained HCT-mono-mIL12 across the tumor interstitium (**Figure 6**, steps 2 and 3), are interpreted as a form of competition between tumor antigen binding and antigen-mediated local clearance (i.e., antigen metabolic turnover) *versus* the intratumoral diffusion mediated by Ab–antigen interactions. This is related to the concept of binding site barrier (7), as previously reported for tumor-targeted Abs (8, 9) and Ab–drug conjugates (35–37). Thus, these steps are governed by the dissociation rate constant (*k*_off_) of the Ab moiety for HER2 antigen: the slower the dissociation rate from the tumor surface antigen, the longer it takes to transport over a given distance (6). The HCT/0.5-mono-mIL12 variant with the highest affinity for HER2 (*K*_D_ ≈ 0.54 nM) was predominantly driven by a ~55 to 162-fold slower dissociation rate (*k*_off_), compared with those of the lower-affinity variants. Therefore, upon extravasation from the blood vessels to the tumor tissues, HCT/0.5-mono-mIL12 tends to tightly bind to the first encountered HER2-expressing cells near the blood vessels due to the slow dissociation rate (*k*_off_ = 0.18 × 10^-3^ s^-1^ or ~93 min), limiting its deeper penetration. Conversely, HCT/130-mono-mIL12 with moderate affinity for HER2 (*K*_D_ ≈ 130 nM) and a much faster *k*_off_ (18.9 × 10^-3^ s^-1^ or ~53 s) can deeply penetrate the distant regions in free form by repetitive cycles of HER2 binding and dissociation within the tumor interstitial space (**Figure 6**, step 2). Another important factor affecting the intratumoral diffusion of HCT-mono-mIL12 is the intracellular internalization rate (*k*_e_) of HER2 receptor compared with the *k*_off_. Prolonged retention of HCT-mono-mIL12 on HER2-expressing cells due to a *k*_off_ that is slower than the *k*_e_ of HER2 on tumor cells can result in depletion *via* HER2-mediated endocytosis, followed by lysosomal degradation (**Figure 6**, step 3), as seen with antitumor Abs (38). The reported *k*_e_ of HER2 on cells ranges from 7.7 × 10^-4^ s^-1^ (~22 min) to 6.67 × 10^-4^ s^-1^ (~25 min) (39). Therefore, when HCT/0.5-mono-mIL12 with a *k*_off_ of ~93 min binds to HER2 receptor, it is predominantly internalized in the cell before its dissociation from HER2, resulting in HER2-mediated clearance and further limiting the intratumoral diffusion (**Figure 6**, step 3). This mechanism supports the observed correlation between the very high affinity of HCT/0.5-mono-mIL12 and its poor tumor penetration. Conversely, HCT/130-mono-mIL12 with a much faster *k*_off_ than *k*_e_ of HER2 dissociates from HER2 before cellular endocytosis, leading to its sustainable extracellular presence and deeper penetration (**Figure 6**, step 3). By contrast, the lowest-affinity HCT/217-mono-mIL12 variant showed the lowest accumulation in the tumor due to loose binding to the tumor cells. Despite its low levels, HCT/217-mono-mIL12 persisted in the TME given that it had the fastest *k*_off_ (29.2 × 10^-3^ s^-1^ or ~34 s), thereby eliciting the tumor infiltration and activation of T cells and exerting substantial antitumor activity. Taken together, our results suggest that an affinity that is too high for the targeted tumor antigen due to a slower *k*_off_ than *k*_e_ for the tumor antigen will restrict the intratumoral diffusion and distribution of immunocytokines *via* the binding site barrier. A much higher dose of a high-affinity binder could increase the probability of more deeply penetrating the tumor tissue (6). However, a higher dose of immunocytokines could be associated with an increased risk of cytokine-mediated systemic toxicity. For this reason, most immunocytokines, including IL12 immunocytokines, are typically administered at very low doses (e.g., 13–20 μg/kg per dose) in clinical trials (15, 40). Considering the results of our study, we recommend selecting Ab-based immunocytokines according to the appropriate tumor antigen-binding kinetics and affinity to balance their preferential accumulation in targeted tumors and intratumoral diffusion, which would otherwise limit the antitumor activity of immunocytokines.

The lack of sufficient functional TILs in the TME is one of the factors leading to poor responses to immunotherapy. The antitumor activity of IL12 is mainly mediated by tumor infiltration and activation of CD4^+^ and CD8^+^ T cells (19). Thus, for potent antitumor effects, deep and broad interstitial distribution of immunocytokines is important to stimulate the population and cytotoxicity of antitumor effector immune cells *in situ* throughout the tumor tissues. We found that the extent of intratumoral diffusion of HCT-mono-mIL12 is strongly correlated with the numbers of CD4^+^ and CD8^+^ T cells and the cytotoxicity of CD8^+^ T cells as well as their even distribution in the TME, suggesting that sufficient presence of IL12 in the TME can induce the tumor infiltration of T cells (41) and/or stimulate the *in situ* proliferation and activation of preexisting T cells to kill tumor cells in the TME. This correlation further clarifies the observed variation in the antitumor activity of HCT-mono-mIL12 according to the anti-HER2 binding kinetic parameters. Treatment of HCT/130-mono-IL12, with the most efficient intratumoral diffusive penetrating activity, increased the number of CD4^+^ and CD8^+^ T cells in the tumors by ~3-fold compared with those found after treatment with higher-affinity variants. Thus, HCT/130-mono-IL12 demonstrated the ability to efficiently promote tumor infiltration and activation of antitumor CD4^+^ and CD8^+^ T cells, thereby increasing the antitumor immunity in the TME (41).

Various molecular architectures of immunocytokines can be designed depending on Ab formats (full-size IgG or Ab fragments) and cytokine forms (monomer, homodimer, or heterodimer) (42). Identification of an optimal format for manufacturing and biological activity is often challenging, particularly for heterodimeric cytokines (43). To date, tumor-targeted IgG-based IL12 immunocytokines have been developed in a bivalent format comprising two molecules of IL12 owing to the symmetric, bivalent architecture of IgG (13, 15, 44). In particular, NHS-IL12, composed of two molecules of IL12 fused to a human IgG1 (NHS76) recognizing DNA/histone complexes found in tumor necrotic portions, delivers IL12 into intratumoral necrotic regions (45). NHS-IL12 is now under early-stage clinical trials against solid tumors (15). In this study, we generated a heterodimeric Fc-based HCT-mono-mIL12 with one molecule of mIL12 based on encouraging findings of the superior antitumor activity of mono-mIL12-Fc to that of bi-mIL12-Fc (19). Our previous study showed that the strong signaling imparted by bi-mIL12-Fc carrying two molecules of mIL12 overstimulated effector T cells, resulting in their conversion to short-lived effector cells, as they diverged from endogenous IL12 signaling (19). In contrast, mono-mIL12-Fc with one molecule of mIL12 triggered weaker signaling of IL12 compared to bi-mIL12-Fc, favoring the generation of functional and protective memory CD8^+^ T cells. Thus, mono-mIL12-Fc outperformed the bivalent bi-mIL12-Fc in terms of enhancing the proliferation and cytotoxic potential of CD8^+^ T cells, and thus the antitumor efficacy in mice bearing large established syngeneic tumors (19). Further, IgG-mono-IL12 would exhibit a longer serum half-life and higher tumor accumulation by avoiding the depletion by IL12R-expressing immune cells during circulation compared with its counterpart with two IL12 molecules, as previously observed with mono-mIL12-Fc (19) and other immunocytokines (46) Accordingly, the format of IgG-mono-IL12 may be superior to its counterpart bearing two molecules of IL12 in terms of induction of potent antitumor immune responses and pharmacokinetics, which remains to be determined in further studies.

In conclusion, our results provide a deeper understanding of the role of anti-HER2 binding kinetics and affinity of HCT-mono-mIL12 in tumor targeting and intratumoral diffusive penetration, thereby determining the tumor infiltration and *in situ* proliferation and activation of tumor-infiltrating effector T cells, and consequently the antitumor activity. Although the highest-affinity HCT/0.5-mono-mIL12 variant can effectively accumulate in tumor tissues and persist to bind target cells, its intratumoral diffusive penetration into solid tumors is extremely limited due to the binding site barrier, resulting in low antitumor activity. Conversely, HCT/130-mono-mIL12 with moderate tumor antigen-binding kinetics and affinity showed less efficient accumulation, but penetrated the targeted tumors more deeply and evenly, thereby exerting more potent antitumor effects. These results using a model of HCT-mono-mIL12 can also be applied to other tumor antigen–target Ab-based immunocytokines. Limitations of this study include the use of a single tumor model and a single dose for anti-HER2 HCT-mono-mIL12 variants. Since the tumor homing and intratumoral penetration of an immunocytokine can be varied depending on the type of tumor antigen and its expression density on the surface as well as the dosing amount, more extensive studies are necessary to generalize the relationship between the tumor antigen binding kinetics of an immunocytokine and the antitumor effects. Finally, our study suggests that in developing immunocytokines for the immunotherapy of solid tumors, fine-tuning of appropriate tumor antigen-binding kinetics and affinity of the tumor–target Ab moiety depending on the tumor antigen expression levels and internalization rate is essential for selective tumor targeting, effective intratumoral diffusion, and eventually achieving maximal antitumor potency without systemic toxicity.

## Data availability statement

The original contributions presented in the study are included in the article/**Supplementary Material**. Further inquiries can be directed to the corresponding author.

## Ethics statement

The animal study was reviewed and approved by Institutional Animal Care and Use Committee of Ajou University.

## Author contributions

Y-SK conceived and designed the experiments. SY, KJ, and J-EK performed the *in vitro* and/or *in vivo* experiments. WK assisted with the biodistribution study. All authors analyzed and interpreted the data. SY, KJ, and Y-SK wrote the manuscript. Y-SK supervised the project. All authors have read and approved the final version of the manuscript.

## Funding

This work was supported by a grant from Dragonfly Therapeutics, Inc.; a grant of the Korea Health Technology R&D Project (HR16C0001) through the Korea Health Industry Development Institute (KHIDI), funded by the Ministry of Health & Welfare; and a grant from the Priority Research Center Program (2019R1A6A1A11051471) through the National Research Foundation of Korea (NRF), funded by the Ministry of Science, ICT & Future Planning, Republic of Korea. The funders had no role in study design; in the collection, analysis and interpretation of data; in the writing of the report; and in the decision to submit the article for publication.

## Acknowledgments

We thank all of the individuals who donated blood samples used in this study.

## Conflict of interest

The authors declare that the research was conducted in the absence of any commercial or financial relationships that could be construed as a potential conflict of interest.

## Publisher’s note

All claims expressed in this article are solely those of the authors and do not necessarily represent those of their affiliated organizations, or those of the publisher, the editors and the reviewers. Any product that may be evaluated in this article, or claim that may be made by its manufacturer, is not guaranteed or endorsed by the publisher.
