# Supplementary material for: Improved intratumoral penetration of IL12 immunocytokine enhances the antitumor efficacy
Source: Front Immunol. 2022 Oct 27;13:1034774. doi: 10.3389/fimmu.2022.1034774 (PMC9667294; doi:10.3389/fimmu.2022.1034774)
Supplement: Supplementary file 1 [file DataSheet_1.pdf]

Supplementary Material

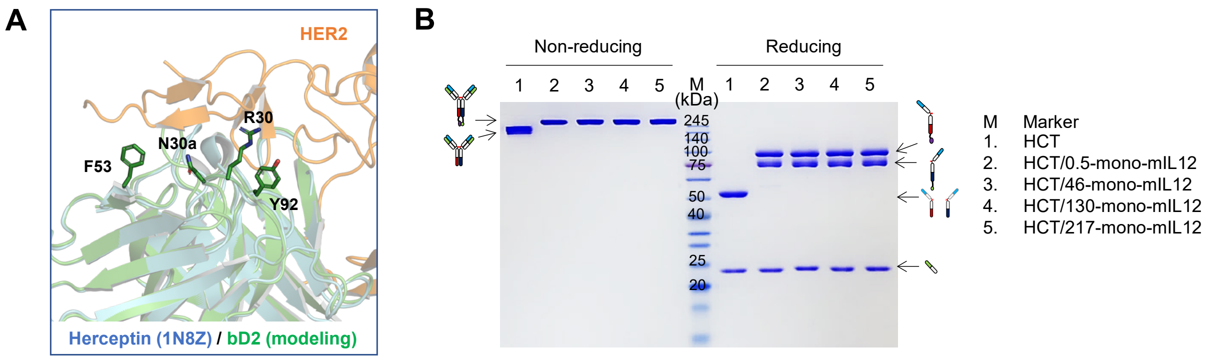

Supplementary Figure 1. Design and characterization of HCT-mono-mIL12 variants. (A) Superposition of the crystal structure (PDB ID: 1N8Z) of Herceptin (cyan) complexed with HER2 antigen (orange) and a modeled structure of bD2 (green) highlighting the mutated residues in the VL-CDRs of bD2. The sequences of VL-CDRs of anti-HER2 affinity variants are shown listed in Fig. 1B. (B) SDS-PAGE analysis of the purified HCT-mono-mIL12 variants. Each protein (5 µg) was analyzed by SDS-PAGE in a 12% gel under non-reducing conditions, and then stained with Coomassie Blue R-250.

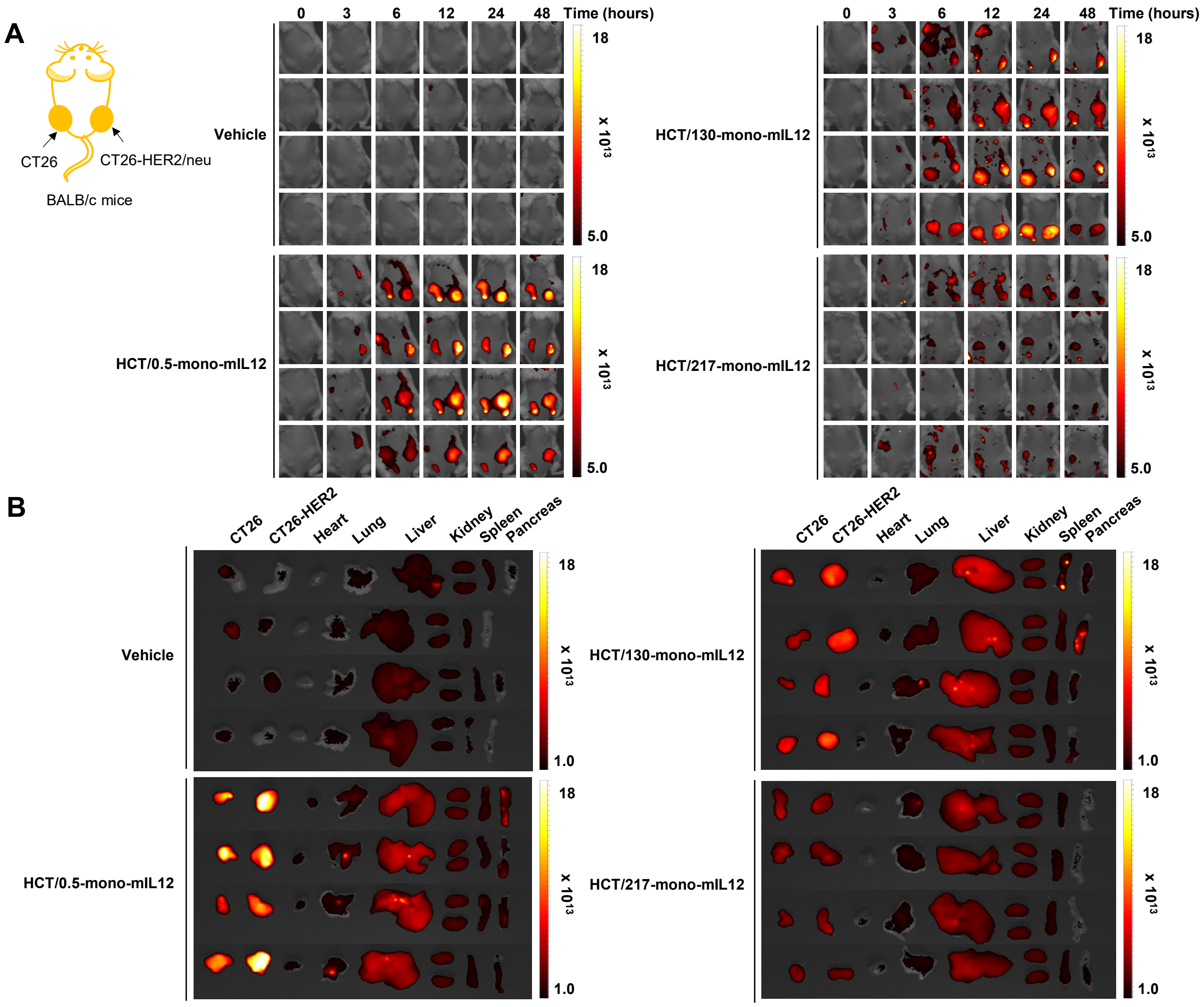

Supplementary Figure 2. Additional images showing biodistribution of DyLight 680-labeled HCT-mono-mIL12 in BALB/c mice bearing dual-flank tumors of CT26 and CT26-HER2/neu, the representative of which is shown in Fig. 2A (A) and Fig. 2B (B). (A) Whole body fluorescence images showing biodistribution of DyLight 680-labeled HCT-mono-mIL12 according to the indicated time after a single i.p. injection (at an equimolar amount of 10 µg rmIL12) into BALB/c mice bearing dual-flank tumors of CT26 (left flank) and CT26-HER2/neu (right flank) at the tumor volume of ~300 mm^3, as described in Fig. 2A.

(B) *Ex vivo* analysis of fluorescence for excised tumors and normal organs 48 h after a single i.p. injection of DyLight 680–labeled HCT-mono-mIL12, as shown in (A). The quantification of fluorescence intensities is shown in Fig. 2B.

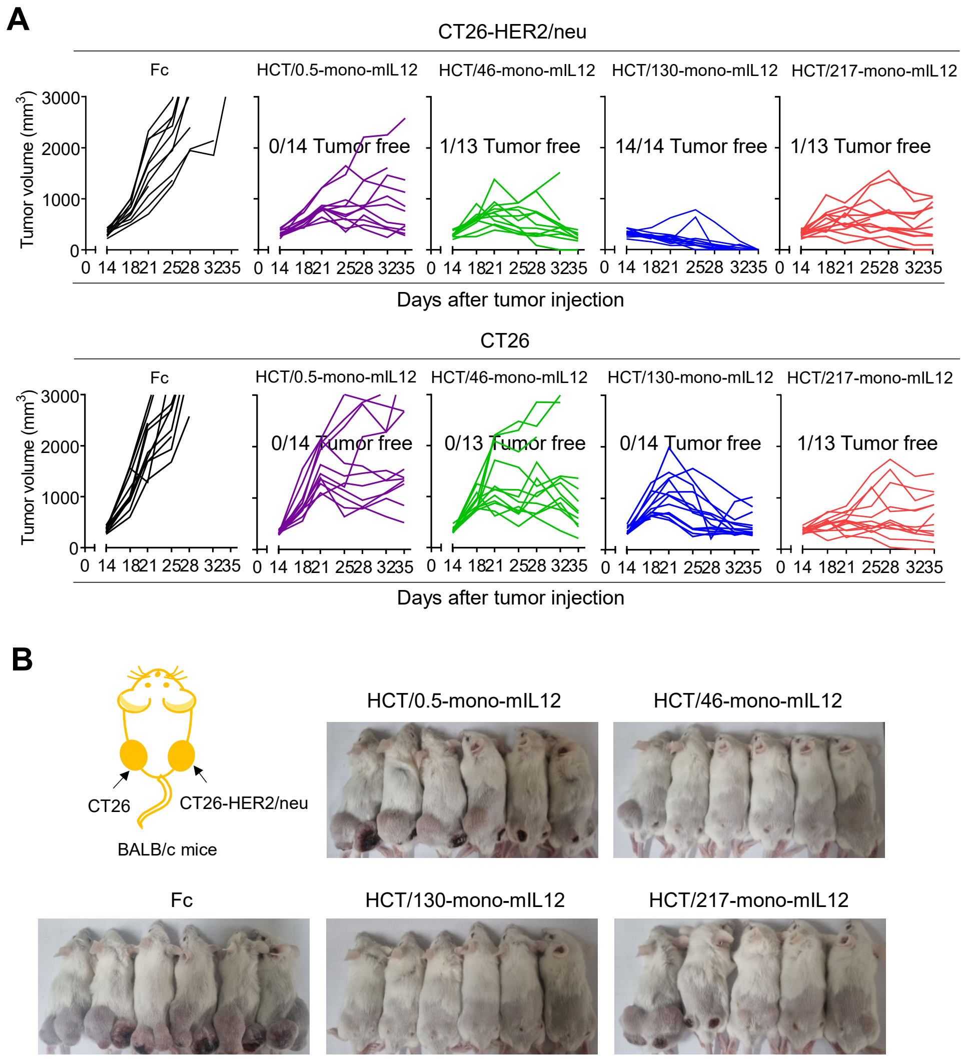

Supplementary Figure 3. Selective tumor accumulation and antitumor activity of HCT-mono-mIL12 requires a threshold anti-HER2 affinity, as described in Fig. 2C-E. (A) Tumor growth profiles of individual mice bearing dual-flank tumors of CT26 (left flank) and CT26-HER2/neu (right flank), treated as described in Fig. 2C. Each curve represents a tumor growth curve, constructed using tumor volume data from each mouse. The number of tumor-free mice per group is shown for each group.
(B) Pictures of TBM (treated as described in Fig. 2C) taken 35 days after tumor inoculation (n = 5-6/group). In (A, B), the data from 2 independent experiments were pooled.

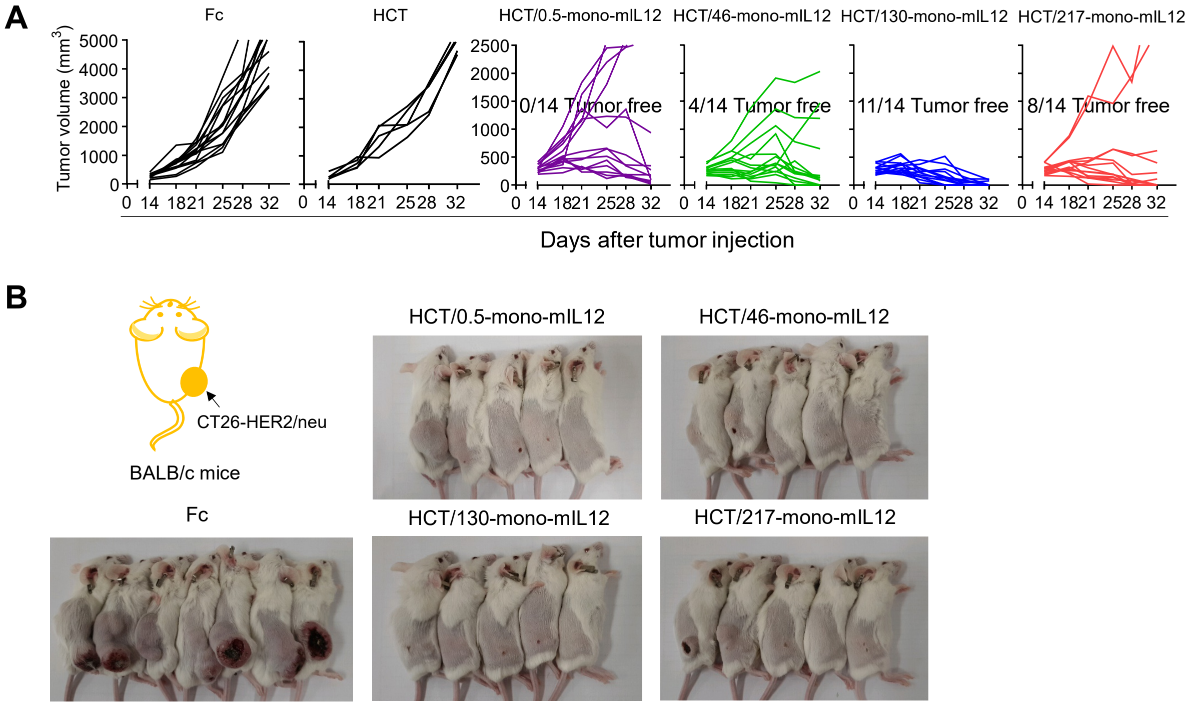

Supplementary Figure 4. *In vivo* antitumor efficacy of HCT-mono-mIL12 varies depending on its anti-HER2 binding kinetics, as described in Fig. 3B. (A) Tumor growth profiles of individual mice bearing a single-flank tumor of CT26-HER2/neu, treated as described in Fig. 3A. Each curve represents a tumor growth curve from each mouse. The number of tumor-free mice per group is shown for each group. (B) Pictures of TBM (treated as described in Fig. 3A) taken 35 days after tumor inoculation (n=5-7/group). In (A, B), the data from 2-3 independent experiments were pooled.
